# Exploring the toxicological network in diabetic microvascular disease

**DOI:** 10.1097/JS9.0000000000002394

**Published:** 2025-04-11

**Authors:** Siyuan Song, Liji Huang, Xiqiao Zhou, Jiangyi Yu

**Affiliations:** a Affiliated Hospital of Nanjing University of Chinese Medicine, Nanjing, Jiangsu Province, China; b Nanjing University of Chinese Medicine, Nanjing, Jiangsu Province, China; c Department of Endocrinology, Jiangsu Province Hospital of Chinese Medicine, Affiliated Hospital of Nanjing University of Chinese Medicine, Nanjing, China

**Keywords:** diabetic kidney disease, diabetic microvascular disease, endocrine disrupting chemicals, network toxicology

## Abstract

**Purpose:**

This study investigates how endocrine-disrupting chemicals contribute to diabetic microvascular disease.

**Methods:**

This study assessed endocrine-disrupting chemical toxicity using PubChem, ProTox 3.0, and ChEMBL. Relevant EDC targets were identified via SwissTargetPrediction and Similarity Ensemble Approach. Gene targets for diabetic microvascular diseases (diabetic kidney disease, retinopathy, and sensory polyneuropathy) were retrieved from CTD, GeneCards, and OMIM. Candidate targets were identified by intersecting EDC and disease-related targets. A protein-protein interaction network was built using STRING to identify hub genes. Functional enrichment analysis was conducted via Metascape. Molecular docking of EDC compounds with hub targets was performed using Discovery Studio and CDOCKER. Hub targets were validated through immunohistochemical staining, single-cell distribution, subcellular localization assays, and gene expression analysis in external HPA and GEO datasets.

**Results:**

A total of 843, 474, and 623 potential toxic targets were identified for diabetic kidney disease, diabetic retinopathy, and diabetic sensory polyneuropathy, respectively. KEGG pathway analysis linked EDC toxicity in diabetic kidney disease to key pathways such as cancer, chemokine signaling, apoptosis, calcium signaling, and drug metabolism (cytochrome P450), with hub targets including EGFR, ALB, MYC, ESR1, and HSP90AA1. Diabetic retinopathy was associated with MAPK, ERBB, NOD-like receptor signaling, and renal cell carcinoma pathways, with ALB, EGFR, MYC, BCL2, and CD4 identified as hub targets. For diabetic sensory polyneuropathy, EDCs may influence chemokine, apoptosis, ERBB, VEGF, and JAK-STAT signaling pathways, with ALB, EGFR, MYC, ESR1, and BCL2 as key targets. Molecular docking confirmed strong binding activity between EDC components and hub targets.

**Conclusion:**

This study offers a theoretical basis for identifying toxic targets and mechanisms by which endocrine-disrupting chemicals contribute to diabetic microvascular diseases.

## Introduction

Endocrine-disrupting chemicals (EDCs) are exogenous substances that interfere with the synthesis, secretion, transport, binding, action, or elimination of endogenous hormones, which regulate homeostasis, reproduction, development, and behavior. This definition underscores the potential of EDCs to disrupt the intricate functions of the endocrine system. Over time, EDCs have been further characterized as “exogenous chemical substances or mixtures that can interfere with any aspect of hormone action”^[^1^]^. Major categories of EDCs include plasticizers, polychlorinated biphenyls, organochlorine pesticides, dioxins, and their analogs. Among them, bisphenols and phthalates are the most prevalent, with bisphenol A (BPA) and bis(2-ethylhexyl) phthalate (DEHP) serving as prototypical examples. Ubiquitous in modern life, EDCs are found in plastic water bottles, food packaging, cosmetics, toys, detergents, and pesticides, raising significant concerns about their long-term health effects^[^2^]^. Epidemiological data suggest that the rising incidence of diabetes, cancer, and infertility over the past two to three decades is, at least in part, attributable to prenatal exposure to EDCs^[^3,4^]^.

**HIGHLIGHT**

- This study offers a theoretical basis for identifying toxic targets and mechanisms by which endocrine-disrupting chemicals contribute to diabetic microvascular diseases.
- This study provides new insights into the pathogenic mechanisms of EDCs in DmiVD (DKD, DR, and DSPN) development.
- Future strategies targeting EDCs, such as developing drugs to eliminate EDCs or inhibit their effects, may offer novel approaches for preventing and treating DmiVD (DKD, DR, and DSPN).

Diabetes mellitus (DM) is a metabolic disorder characterized by chronic hyperglycemia and has emerged as a major global public health concern. Projections indicate that by 2040, the global prevalence of diabetes among adults will rise to 10.4%, affecting approximately 642 million individuals^[^5,6^]^. Diabetic microvascular disease (DMiVD), encompassing diabetic kidney disease (DKD), diabetic retinopathy (DR), and distal symmetrical polyneuropathy (DSPN), constitutes a primary risk factor for end-stage renal disease, blindness, limb amputation, and foot ulcers, particularly in developed countries^[^7^]^. Early identification and management of microvascular complications are critical, as up to 25% of newly diagnosed type 2 diabetes mellitus patients already exhibit one or more complications at the time of diagnosis^[^8,9^]^. Recent studies^[^10^]^ suggest that, beyond conventional risk factors such as obesity, genetic predisposition, and lifestyle, exposure to EDCs is also strongly implicated in DMiVD pathotargetsis. For instance, BPA, a widely used industrial chemical, serves as a plasticizer, heat stabilizer, and flame retardant in the production of plastic packaging, baby bottles, and dental fillings. Given its extensive use, BPA exposure occurs primarily through direct contact with consumer products, raising significant concerns regarding its potential role in diabetes-related vascular complications.

BPA increases DM risk by disrupting pancreatic β-cell function and reducing insulin sensitivity in organs like skeletal muscle^[^11^]^. It impairs insulin signaling, glucose oxidation, and glycogen content through downregulation of the PKB/Akt pathway and insulin receptor expression^[^12^]^. Heavy metals such as cadmium, lead, mercury, chromium, and tin also disrupt endocrine function and accumulate in the food chain, posing health risks. Low-dose BPA binds estrogen receptor β, affecting ion channels and impairing insulin secretion^[^13^]^, contributing to its diabetogenic effects. Disruption of β-cell activity is a key mechanism of BPA’s toxicity in DM. Yang *et al*^[^14^]^ found a positive correlation between manganese exposure and hyperglycemia. Similarly, Little *et al*^[^15^]^ assessed chronic cadmium exposure in a population residing near a metal smelter and found a strong association with an increased risk of DM and DMiVD. Furthermore, Li *et al*^[^16^]^ demonstrated that oxidative stress-induced mitochondrial dysfunction and impaired ATP production may underlie the inhibitory effects of inorganic arsenic and its methylated metabolites on glucose-stimulated insulin secretion in pancreatic β-cells, both *in vivo* and *in vitro*. Minimizing EDC exposure is crucial to reduce potential harm and alleviate DMiVD severity. However, the exact toxicological mechanisms by which EDCs contribute to DM and DMiVD pathogenesis are not well understood. To address this, we included 12 major EDCs in this study (Supplemental Digital Content Table 1, available at: http://links.lww.com/JS9/E63) for further investigation.

Network toxicology investigates potential toxicity mechanisms by constructing multi-component regulatory networks and identifying toxic targets, thereby elucidating the molecular basis of toxicity^[^17,18^]^. Molecular docking is a computational simulation technique widely used in drug design, bioinformatics, and molecular biology. It predicts interactions between small molecules (ligands) and biological macromolecules (such as proteins, DNA, or RNA, referred to as receptors). Molecular docking plays a crucial role in studying drug targets, screening potential therapeutic compounds, and optimizing lead compounds^[^19^]^.

In this study, we employ network toxicology to analyze the toxic effects of EDCs on DMiVD. Additionally, molecular docking technology is utilized to preliminarily validate key components and hub targets of EDC-induced toxicity. This approach aims to provide a foundational reference for future research on the underlying mechanisms of EDC toxicity.

## Method

### Toxicity validation of EDCs

The SMILES sequences of 12 selected EDCs (Supplemental Digital Content Figure 1, available at: http://links.lww.com/JS9/E60) were retrieved from the PubChem database (https://pubchem.ncbi.nlm.nih.gov/). These sequences were then input into ProTox 3.0 (https://tox.charite.de/protox3/) and ChEMBL (https://www.ebi.ac.uk/chembl/) for toxicity prediction and analysis.

### Retrieval of EDC targets

The SMILES structures of the selected EDCs, obtained from the PubChem database, were imported into SwissTargetPrediction (http://www.swisstargetprediction.ch/) and the Similarity Ensemble Approach (SEA) (https://sea.bkslab.org/)^[^20^]^ databases, the target species was set to Homo sapiens to ensure relevance to human biology. These platforms were used to predict, screen, and confirm the potential molecular targets of EDCs.

### Retrieval of DMiVD-related gene targets

To identify gene targets associated with DmiVD (DKD, DR, and DSPN), the keywords “diabetic kidney disease”, “diabetic retinopathy”, and “distal symmetrical polyneuropathy” were used to search the CTD (https://ctdbase.org/), GeneCards (https://www.genecards.org/), and OMIM (https://omim.org/) databases. The retrieved gene targets were then compiled for further analysis.

### Identification of common targets between EDCs and DMiVD

Using the online tool Venny 2.1.0 (https://bioinfogp.cnb.csic.es/tools/venny/), the predicted targets of 12 major EDCs were mapped against the gene targets associated with DMiVD. The intersection of these datasets identified candidate targets through which EDCs may exert toxic effects on DMiVD. A Venn diagram was generated to visualize the overlap.

### Construction of the protein-protein interaction network

To further analyze the interactions between the targets of 12 major EDCs and DMiVD-related targets, the intersecting target set was imported into the STRING database (https://string-db.org/) to construct a toxicity-related protein-protein interaction (PPI) network. The resulting PPI network was then imported into Cytoscape 3.9.0, where the MCODE plugin was used to perform clustering analysis of key modules based on the molecular complex detection algorithm. Hub targets were identified by intersecting the key targets from the MCODE-identified core modules with the top-ranked targets predicted by CytoHubba^[^21^]^.

### GO biological process and KEGG pathway enrichment analysis

Metascape^[^22^]^ (https://metascape.org/gp/) integrates over 40 bioinformatics databases, offering comprehensive tools for biological pathway enrichment analysis, protein interaction network analysis, and extensive gene annotation. Gene Ontology (GO) enrichment analysis categorizes gene functions into three main domains: Molecular function (MF), biological process (BP), and cellular components (CC), providing a multi-dimensional characterization of gene roles^[^23^]^. Meanwhile, the Kyoto Encyclopedia of Targets and Genomes (KEGG) pathway enrichment database integrates genomic, chemical, and system-level functional data to establish links between genetic information and biological functions, elucidating the genetic and biochemical foundations of life processes.

To investigate the toxicological mechanisms of EDCs, the hub targets were imported into the Metascape database. GO and KEGG enrichment analyses were performed by inputting the target gene list, selecting Homo sapiens as the species, and setting thresholds of *P* < 0.05 and minimum enrichment value = 1.5. The BPs and metabolic pathways were then visualized to identify the key signaling pathways involved in EDC-induced toxicity and to explore their potential mechanisms.

### Molecular docking analysis

Discovery Studio, a specialized molecular simulation software for life sciences, is widely used in drug discovery and biomacromolecule computational modeling, particularly for studying protein and antibody structures.

In this study, EDCs were used as ligands for molecular docking. The top five hub targets with the highest degree values from the PPI network were selected as receptors. The 3D structures of EDCs were obtained from the PubChem database and saved in SDF format. The CAS numbers of the core toxic targets were used to retrieve their 3D structures from the PDB database (http://www.rcsb.org/), with the species set to Homo sapiens and resolution optimized between 0 and 3.0 Å. Using Discovery Studio 2019, receptor proteins were prepared by adding hydrogen atoms, removing water molecules, and simulating missing loop regions. Binding pockets were then predicted. Ligands were also processed, including hydrogenation and conformational isomer calculations. Molecular docking was performed using the CDOCKER algorithm to predict potential binding modes between key compounds and hub targets. The CDOCKER interaction energy was calculated, where a higher interaction energy indicates a stronger binding affinity between the ligand and target protein.

### Validation of the hub gene expression in databases

By utilizing the HPA database (https://www.proteinatlas.org/), the immunohistochemical (IHC) staining results of the hub targets in different tissues were validated. The staining intensity, positive cell types, and tissue distribution were observed to assess gene expression levels. Single-cell distribution analysis was conducted to examine gene expression in various cell types (e.g. endocrine cells, epithelial cells), and UMap visualization was used to analyze the distribution characteristics of the gene across different cell populations. Additionally, subcellular localization validation was performed using fluorescence microscopy images to observe protein localization in specific cellular compartments such as the nucleus, cytoplasm, mitochondria, and endoplasmic reticulum.

The gene expression datasets related to DKD, DR, and DSPN-GSE30528, GSE60439, and GSE95849-were obtained from the GEO database (https://www.ncbi.nlm.nih.gov/geo/). The GSE30528 dataset comprises 9 DKD samples and 13 normal controls, while GSE60439 includes 6 DR samples and 3 normal controls. Similarly, GSE95849 consists of six DSPN samples and six normal controls.

## Results

### Effects of EDCs on DKD

**Figure 1.**
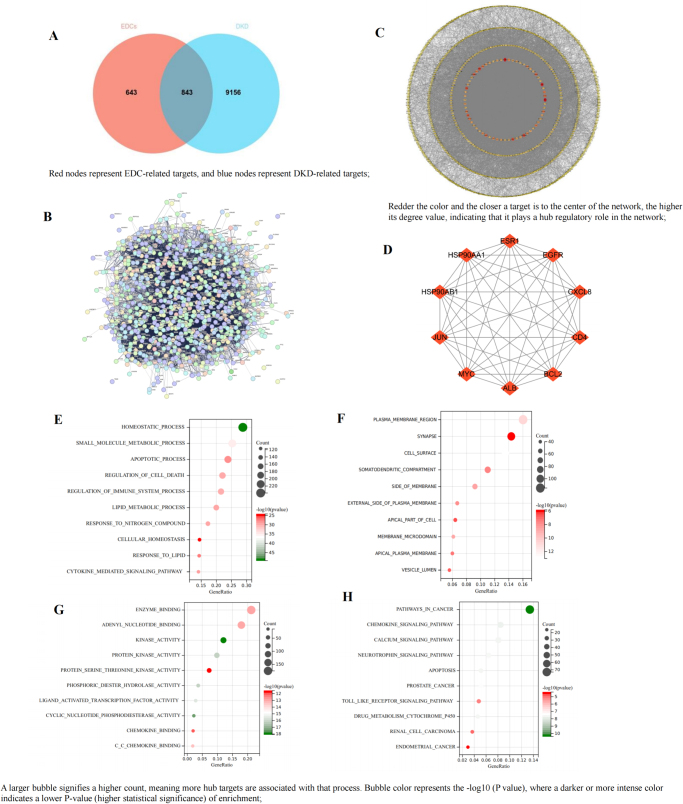
Association analysis between EDCs and DKD. (A) Venn diagram of EDCs targets and DKD-related targets; (B) PPI network of the intersection targets through STRING database; (C) PPI network of the intersection targets through Cytoscape 3.9.0; (D) top 10 hub targets through MCODE and CytoHubba plugin; (E) biological process analysis of hub targets; (F) cellular components analysis of hub targets; (G) molecular function analysis of hub targets; (H) KEGG analysis of hub targets.

The targets of 12 major EDCs were predicted using SwissTargetPrediction and SEA databases. After merging and removing duplicates, 1486 unique EDC-related targets were identified. DKD-related gene targets were retrieved from CTD, GeneCards, and OMIM, yielding 9999 unique targets after filtering. Using Venny 2.1.0, 843 overlapping targets were identified (Fig. 1A), representing potential toxic targets through which EDCs may contribute to DKD pathogenesis.

To analyze these common targets, a PPI network was constructed by importing the 843 shared targets into the STRING database (Fig. 1B) with parameters set to “Multiple proteins,” Homo sapiens, and a high-confidence minimum interaction score. This resulted in a network of 829 nodes and 13 999 edges (Fig. 1C), highlighting the complex interactions between EDC-related toxic targets and DKD pathophysiology.

Since proteins in the PPI network interact with each other, it is represented as an undirected graph. High-density regions, or modules, are biologically significant clusters^[^24^]^. To further investigate EDC-induced toxicity mechanisms in DKD, the network was imported into CytoScape 3.9.0, where MCODE and CytoHubba plugins were used for clustering analysis. The top 10 hub targets were identified based on degree values (Fig. 1D), as these are likely to play key roles in EDC-induced DKD toxicity.

Using the Metascape platform, GO and KEGG pathway enrichment analyses were performed on the 843 candidate targets associated with EDC-induced DKD toxicity. The GO analysis revealed that in terms of BP, the candidate targets are primarily involved in homeostasis, apoptosis, and lipid metabolism, while in CC, they are mainly associated with synapses, plasma membrane regions, and the cell surface. Regarding MF, the targets are predominantly related to enzyme binding, kinase activity, and adenyl nucleotide binding. The KEGG pathway enrichment analysis identified the top 10 pathways based on *P*-values, with the most significant ones being pathways in cancer, chemokine signaling, apoptosis, calcium signaling, and drug metabolism via cytochrome P450. These results provide valuable insights into the potential mechanisms by which EDCs contribute to DKD toxicity, highlighting key BPs and pathways involved in disease progression.

**Figure 2.**
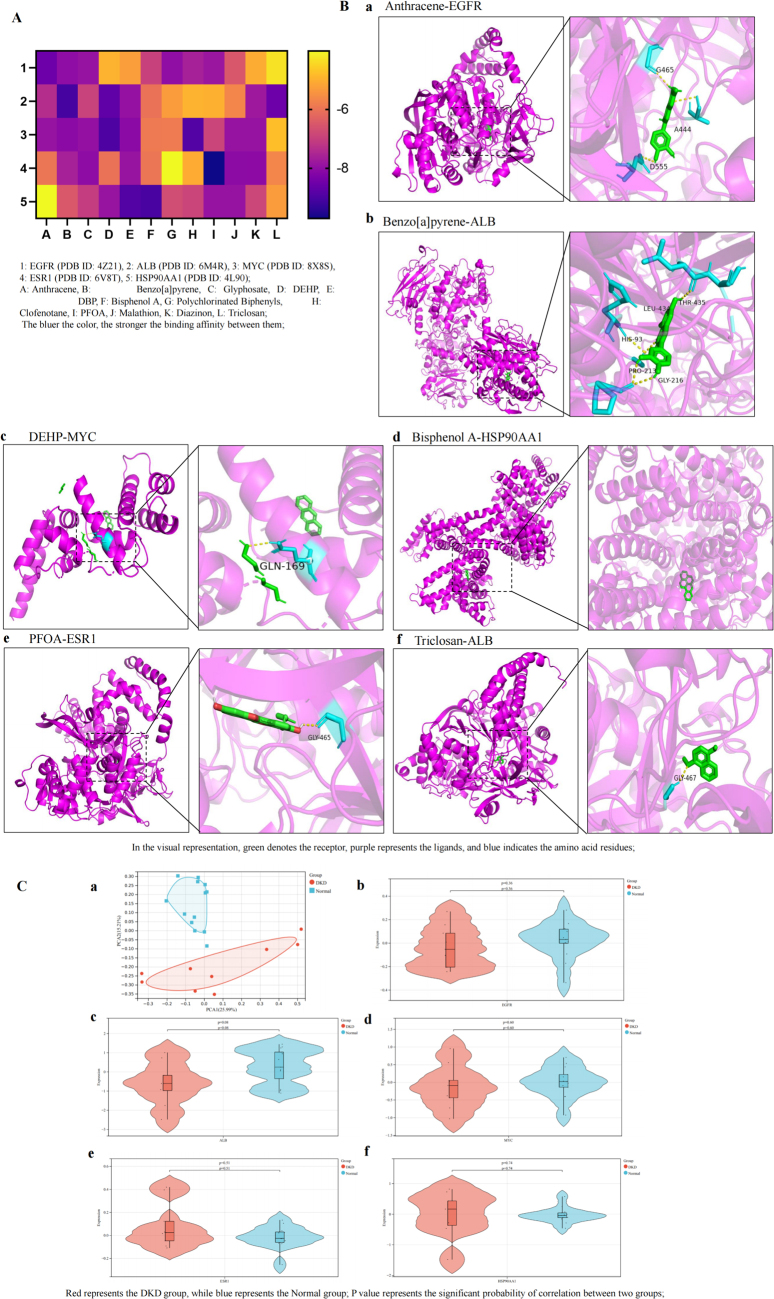
Molecular docking between 12 types of EDCs and the hub targets of DKD. (A) Heatmap of molecular docking between 12 types of EDCs and the top 5 hub targets; (B) molecular docking results with the lowest binding energy were selected for visualization using PyMOL; a, Anthracene-EGFR; b, Benzo[a]pyrene-ALB; c, DEHP-MYC; d, Bisphenol A-HSP90AA1; e, PFOA-ESR1; f, Triclosan-ALB; (C) validation of the hub gene expression in databases; a, PCA map of DKD and normal groups; b, expression of EGFR between the two groups; c, expression of ALB between the two groups; d, expression of MYC between the two groups; e, expression of ESR1 between the two groups; f, expression of HSP90AA1 between the two groups.

The molecular docking heatmap results show that EDCs have strong binding affinities with the top five hub targets (Fig. 2A), including EGFR (PDB ID: 4Z21), ALB (PDB ID: 6M4R), MYC (PDB ID: 8X8S), ESR1 (PDB ID: 6V8T), and HSP90AA1 (PDB ID: 4L90). The top six molecular docking results with the lowest binding energy were selected for visualization using PyMOL^[^25^]^. Notably, the interaction between Anthracene and EGFR involves the G465, D555, and A444 residues, while Benzo[a]pyrene interacts with the THR-435, LEU-434, HIS-93, PRO-213, and GLY-216 residues of ALB. DEHP binds with the GLN-169 residues of MYC, PFOA interacts with the GLY-465 residues of ESR1, and Triclosan interacts with the GLY-467 residues of ALB (Fig. 2B).

Validation through the HPA database showed that the hub targets associated with EDC-induced DKD toxicity-EGFR, MYC, and ALB were primarily located in tubules, with no detection in glomeruli. HSP90AA1 and ESR1 were not detected in either tubules or glomeruli (Supplemental Digital Content Figure 2A, available at: http://links.lww.com/JS9/E61). Single-cell distribution analysis revealed their presence in endocrine tissues (Supplemental Digital Content Figure 2B, available at: http://links.lww.com/JS9/E61). Subcellular localization analysis indicated that EGFR was mainly found in the plasma membrane, ALB in the endoplasmic reticulum, MYC and ESR1 in the nucleoplasm, and HSP90AA1 in the cytosol (Supplemental Digital Content Figure 2C, available at: http://links.lww.com/JS9/E61).

In the GSE30528 dataset, PCA analysis of the hub targets related to EDC-induced DKD toxicity showed a clear distinction between the normal and DKD groups, with PCA1 explaining 25.99% of the variance and PCA2 accounting for 15.21%. EGFR, ALB, and MYC were downregulated in DKD, while ESR1 and HSP90AA1 were upregulated, though the differences were not statistically significant (Fig. 2C).

### Effects of EDCs on DR

**Figure 3.**
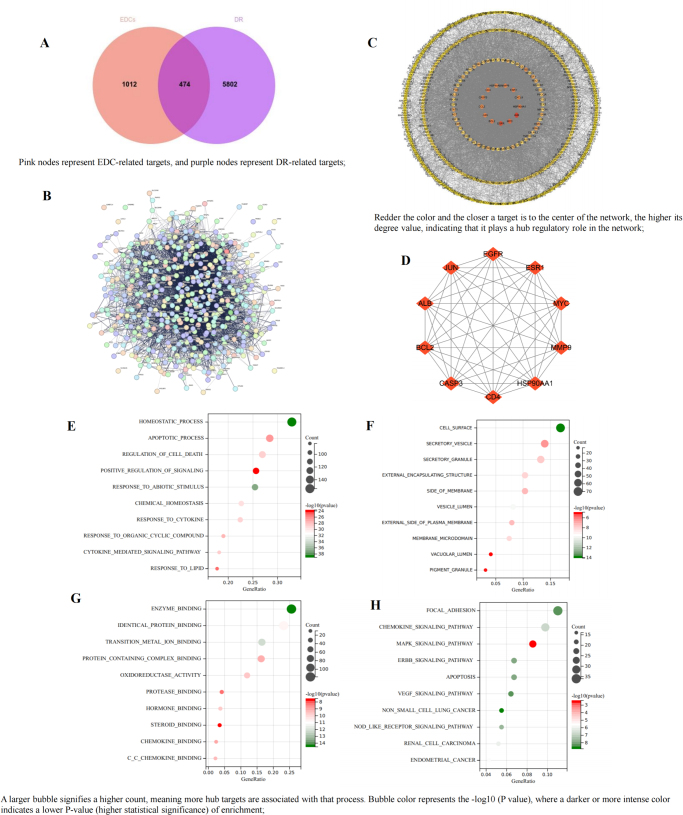
Association analysis between EDCs and DR. (A) Venn diagram of EDCs targets and DR-related targets; (B) PPI network of the intersection targets through STRING database; (C) PPI network of the intersection targets through Cytoscape 3.9.0; (D) top 10 hub targets through MCODE and CytoHubba plugin; (E) biological process analysis of hub targets; (F) cellular components analysis of hub targets; (G) molecular function analysis of hub targets; (H) KEGG analysis of hub targets.

A total of 6276 DR-related gene targets were retrieved from CTD, GeneCards, and OMIM databases. Venny 2.1.0 identified 474 overlapping targets between EDC-associated and DR-related targets (Fig. 3A), representing potential mechanisms through which EDCs contribute to DR.

A PPI network was constructed by importing the 474 overlapping targets into the STRING database (Fig. 3B), with parameters set to “Multiple proteins,” Homo sapiens, and a “High confidence” interaction score. This resulted in a network of 465 nodes and 6519 edges (Fig. 3C), highlighting the complex interactions between EDC-related toxic targets and DR pathophysiology. The top 10 hub targets were selected based on degree values (Fig. 3D), as they are expected to play key roles in EDC-induced DR toxicity.

Using the Metascape platform, GO and KEGG pathway enrichment analyses were performed on the 474 candidate targets related to EDC-induced DR toxicity. The GO analysis showed that in BP, the targets are mainly involved in apoptosis, regulation of cell death, and positive regulation of signaling (Fig. 3E). In CC, they are associated with secretory vesicles, external encapsulating structures, and membrane sides (Fig. 3F). In MF, they are linked to identical protein binding, protease binding, and transition metal ion binding (Fig. 3G). The KEGG pathway analysis identified the top 10 pathways based on P-values, with significant ones including MAPK, ERBB, NOD-like receptor signaling, and renal cell carcinoma pathways (Fig. 3H). These results offer insights into the mechanisms by which EDCs contribute to DR toxicity, emphasizing key BPs and pathways involved in disease progression.

**Figure 4.**
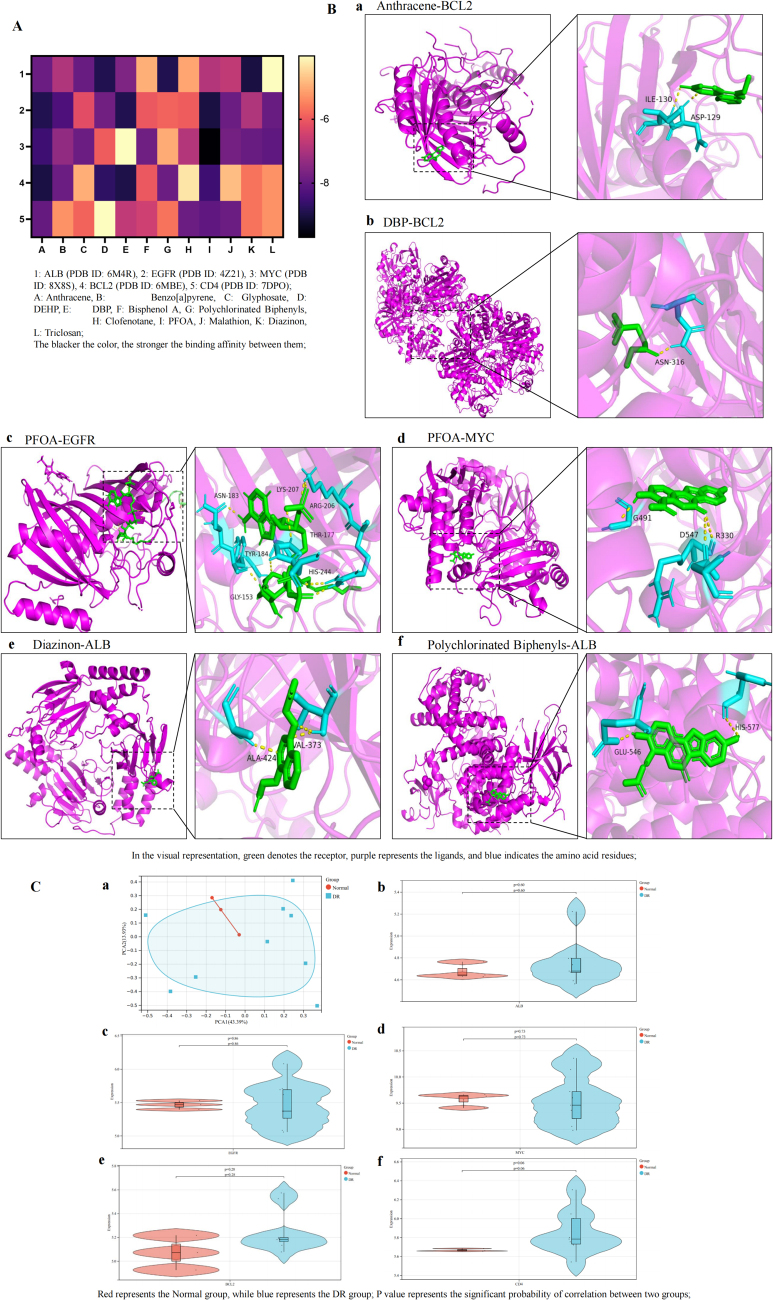
Molecular docking between 12 types of EDCs and the hub targets of DR. (A) Heatmap of molecular docking between 12 types of EDCs and the top 5 hub targets; (B) molecular docking results with the lowest binding energy were selected for visualization using PyMOL; a, Anthracene-BCL2; b, DBP-BCL2; c, PFOA-EGFR; d, PFOA-MYC; e, Diazinon-ALB; f, polychlorinated biphenyls-ALB; (C) validation of the hub gene expression in databases; a, PCA map of DR and normal groups; b, expression of ALB between the two groups; c, expression of EGFR between the two groups; d, expression of MYC between the two groups; e, expression of BCL2 between the two groups; f, expression of CD4 between the two groups.

The molecular docking heatmap results show that EDCs have strong binding affinities with the top five hub targets (Fig. 4A), including ALB (PDB ID: 6M4R), EGFR (PDB ID: 4Z21), MYC (PDB ID: 8X8S), BCL2 (PDB ID: 6MBE), and CD4 (PDB ID: 7DPO). The top six molecular docking results with the lowest binding energy were visualized using PyMOL. Notably, Anthracene interacts with the ILE-130 and ASP-129 residues of BCL2, DBP binds to the ASN-316 residue of BCL2, and PFOA interacts with the ASN-183, LYS-207, ARG-206, THR-177, HIS-244, TYR-184, and GLY-153 residues of EGFR. Additionally, PFOA interacts with the G491, D547, and R330 residues of MYC. Diazinon interacts with the ALA-424 and VAL-373 residues of ALB, while polychlorinated biphenyls interact with the GLU-546 and HIS-577 residues of ALB (Fig. 4B).

HPA database validation showed that BCL2 and CD4, core targets of EDC-induced DR toxicity, were found in endocrine tissues (Supplemental Digital Content Figure 3A, available at: http://links.lww.com/JS9/E62). BCL2 localized to the mitochondria, and CD4 to the plasma membrane (Supplemental Digital Content Figure 3B, available at: http://links.lww.com/JS9/E62). Other targets were validated in Effects of EDCs on DKD.

In the GSE60439 dataset, PCA analysis showed a clear separation between normal and DR groups, with PCA1 explaining 43.39% and PCA2 13.93% of the variance. ALB, EGFR, MYC, BCL2, and CD4 were upregulated in DR patients, though not statistically significant (Fig. 4C).

### Effects of EDCs on DSPN

**Figure 5.**
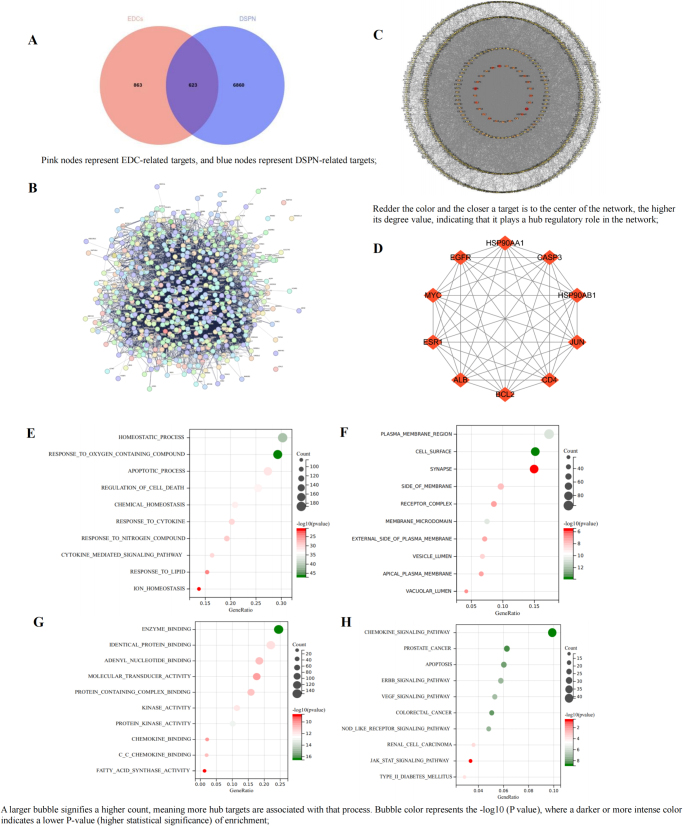
Association analysis between EDCs and DSPN. (A) Venn diagram of EDCs targets and DSPN-related targets; (B) PPI network of the intersection targets through STRING database; (C) PPI network of the intersection targets through Cytoscape 3.9.0; (D) top 10 hub targets through MCODE and CytoHubba plugin; (E) biological process analysis of hub targets; (F) cellular components analysis of hub targets; (G) molecular function analysis of hub targets; (H) KEGG analysis of hub targets.

Gene targets related to DSPN were collected from the CTD, GeneCards, and OMIM databases. After filtering and removing duplicates, 7483 relevant gene targets were identified. Venny 2.1.0 analysis showed 623 common targets (Fig. 5A), predicted as toxic targets potentially contributing to DSPN pathogenesis through EDC exposure.

A PPI network was constructed by importing the 623 overlapping targets into the STRING database (Fig. 5B), with settings for “Multiple proteins,” Homo sapiens, and “High confidence” for the minimum interaction score. The analysis generated a network with 613 nodes and 9241 edges (Fig. 5C), illustrating complex interactions between EDC-related toxic targets and DSPN pathophysiology. Based on degree values, the top 10 hub targets were selected (Fig. 5D), expected to play key roles in the progression of EDC-induced DSPN toxicity.

The Metascape platform performed GO and KEGG analysis on 623 candidate targets related to EDC toxicity in DSPN. GO analysis shows these targets are mainly involved in homeostasis, response to oxygen compounds, and apoptosis (BP); plasma membrane, cell surface, and synapse (CC); and enzyme binding, protein binding, and nucleotide binding (MF). KEGG analysis revealed top pathways like chemokine signaling, apoptosis, ERBB, VEGF, JAK-STAT, and type II diabetes. These findings provide insights into the BPs and pathways linked to DSPN toxicity.

**Figure 6.**
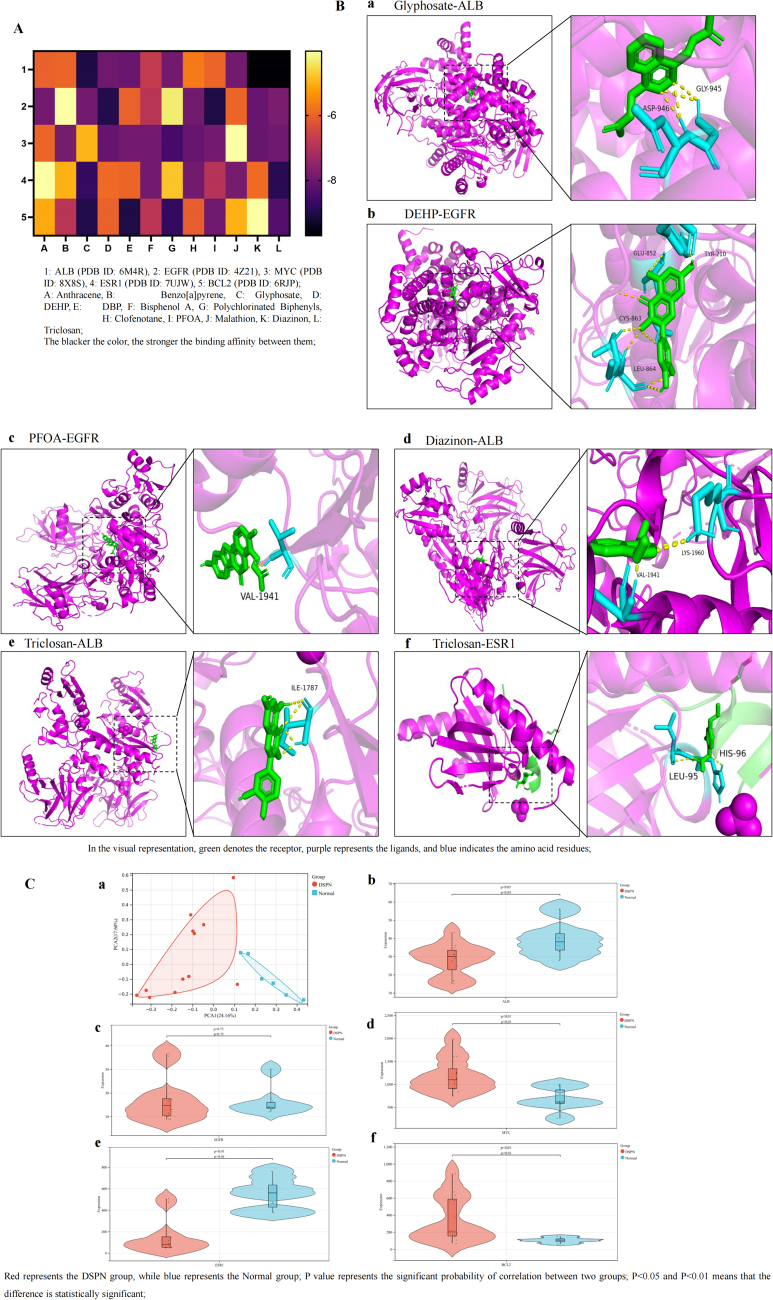
Molecular docking between 12 types of EDCs and the hub targets of DSPN. (A) Heatmap of molecular docking between 12 types of EDCs and the top 5 hub targets; (B) molecular docking results with the lowest binding energy were selected for visualization using PyMOL; a, Glyphosate-ALB; b, DEHP-EGFR; c, PFOA-EGFR; d, Diazinon-ALB; e, Triclosan-ALB; f, Triclosan-ESR1; (C) validation of the hub gene expression in databases; a, PCA map of DSPN and normal groups; b, expression of ALB between the two groups; c, expression of EGFR between the two groups; d, expression of MYC between the two groups; e, expression of ESR1 between the two groups; f, expression of BCL2 between the two groups.

The molecular docking heatmap shows strong binding affinities between EDCs and the top five hub targets: ALB (PDB ID: 6M4R), EGFR (PDB ID: 4Z21), MYC (PDB ID: 8X8S), ESR1 (PDB ID: 7UJW), and BCL2 (PDB ID: 6RJP) (Fig. 6A). The six lowest binding energy interactions were visualized using PyMOL. Glyphosate interacts with GLY-945 and ASP-946 of ALB, DEHP binds to EGFR at GLU-852, TYR-210, CYS-863, and LEU-864. PFOA binds to VAL-1941 of EGFR, Diazinon interacts with ALB at LYS-1960 and VAL-1941, and Triclosan engages with ILE-1787 of ALB and LEU-95, HIS-96 of ESR1 (Fig. 6B).

The hub targets linked to EDC-induced DSPN toxicity were validated in Effects of EDCs on DKD and Effects of EDCs on DR. In the GSE95849 dataset, PCA analysis showed a clear distinction between the normal and DSPN groups, with PCA1 accounting for 24.16% and PCA2 for 17.60% of the variance. EGFR was upregulated in DSPN patients, but not significantly. ALB and ESR1 were downregulated, while MYC and BCL2 were upregulated, with significant differences (*P* < 0.05) (Fig. 6C).

## Discussion

The toxicity of EDCs was initially assessed using data from PubChem, ProTox 3.0, and ChEMBL. Relevant EDC targets were identified via SwissTargetPrediction and SEA platforms. Gene targets related to DmiVD (DKD, DR, and DSPN) were sourced from CTD, Genecards, and OMIM. An intersection of EDC and DmiVD targets formed the PPI network. GO and KEGG enrichment analyses were performed using the Metascape platform. Molecular docking of EDCs with hub targets was conducted using Discovery Studio and the CDOCKER algorithm. IHC staining results were validated with the HPA database, and the expression of hub targets was further confirmed in GEO datasets.

EDCs are substances that interfere with hormone synthesis, secretion, transport, and metabolism, impacting homeostasis and development. They affect reproductive, neurological, and immune systems and are commonly found in everyday life due to industrial development^[^26,27^]^. Humans are easily exposed to EDCs, posing health risks. As awareness grows, many toxic EDCs are now tightly regulated^[^28^]^. However, due to their persistence, bioaccumulation, and ability to cross barriers like the blood-brain and placental barriers, long-term exposure to low EDC concentrations remains a significant threat to human and future generations’ health^[^29,30^]^. This study aims to explore how EDCs contribute to DmiVD (DKD, DR, and DSPN) development.

KEGG pathway enrichment analysis indicates that the potential toxicity of EDCs in DKD pathogenesis is closely linked to key biological pathways, including cancer pathways, chemokine signaling, apoptosis, calcium signaling, and drug metabolism via cytochrome P450^[^31^]^. The oxidative stress pathway, which causes lipid peroxidation in pancreatic β-cells and disrupts their normal function, is a major factor in DKD onset and progression. High glucose levels in DKD can alter CYP450 expression, increasing ROS production and worsening oxidative stress-related damage^[^32^]^. EDCs, like BPA, are believed to worsen kidney damage by inducing oxidative stress and inflammation, as studies show they promote ROS generation, impairing renal tubular cells and contributing to DKD progression^[^33^]^. The MAPK, ERBB signaling pathway, NOD-like receptor signaling pathway, and renal cell carcinoma are involved in the pathogenesis of DR. In a diabetic environment, hyperglycemia stimulates retinal microvascular endothelial cells, causing excessive activation of the MAPK signaling pathway, particularly JNK and p38. This activation induces the release of pro-inflammatory cytokines, such as TNF-α, IL-1β, IL-6, and MCP-1, which worsen retinal inflammation. Persistent inflammation disrupts the blood-retinal barrier (BRB), leading to vascular leakage and macular edema, thus accelerating DR progression^[^34^]^. Aberrant ERBB receptor signaling may impair the function of retinal ganglion cells and Müller cells, accelerating retinal neuronal damage and apoptosis. ERBB4 is crucial for neuroprotection, and its dysregulated expression may contribute to retinal dysfunction in DR patients. Metabolic disturbances in diabetes, such as hyperglycemia and the accumulation of advanced glycation end-products, release endogenous danger-associated molecular patterns, which activate the NLRP3 inflammasome, triggering chronic retinal inflammation. This chronic inflammation disrupts the BRB, promotes neovascularization, and contributes to the onset and progression of DR^[^35^]^. EDCs may contribute to DSPN development by modulating pathways such as chemokine signaling, apoptosis, ERBB signaling, VEGF signaling, and JAK-STAT signaling. The binding of ERBB2 and ERBB3 receptors with nerve growth factors and other neurotrophic factors aids in neuronal repair and regeneration. In diabetes, dysregulation of the ERBB pathway can impair neuronal repair, exacerbating damage and promoting neuropathy progression^[^36^]^. These findings provide insights into the potential mechanisms by which EDCs contribute to DmiVD toxicity, highlighting key BPs and pathways involved in DKD, DR, and DSPN.

EGFR, ALB, MYC, ESR1, HSP90AA1, BCL2, and CD4 were identified the hub targets in the process of EDCs-induced DmiVD toxicity. ALB is a common biomarker for DKD, and changes in plasma albumin levels are closely associated with the onset of diabetic complications, particularly renal dysfunction^[^37^]^. MYC is a transcription factor involved in cell proliferation, metabolic regulation, and anti-apoptotic processes. In diabetes, the overexpression of MYC may promote inflammatory responses and cell proliferation, thereby accelerating damage to the kidneys and vasculature^[^38^]^. ESR1, one of the estrogen receptors, regulates various BPs. Studies have shown that ESR1 is associated with the development of cardiovascular diseases and retinal complications in diabetes, particularly in female diabetic patients. Activation of ESR1 may impact vascular health and neovascularization^[^39^]^. HSP90AA1 is a molecular chaperone protein involved in cellular stress responses and protein folding. HSP90AA1 promotes tissue damage induced by diabetes through its stress response and antioxidant functions, thereby increasing the risk of diabetic complications, particularly in the kidneys and retina^[^40^]^. BCL2 is an anti-apoptotic protein that regulates cell survival. In diabetes, the overexpression of BCL2 may inhibit apoptosis, making cells more resistant to oxidative stress and inflammatory responses, which can lead to abnormal cell survival in pathological conditions^[^41^]^. CD4+ T cells are closely associated with the chronic inflammation induced by diabetes. They may exacerbate diabetic complications by promoting the secretion of inflammatory mediators^[^42^]^.

Our research highlights the significant role of EDCs in the onset and progression of DmiVD, likely through multiple pathways affecting metabolism, immunity, and inflammation. Developing therapeutic strategies targeting EDCs is essential. First, targeted therapies based on the hub targets we identified, involved in insulin signaling, lipid metabolism, inflammation, and oxidative stress, could help manage metabolic disturbances in DM. Drugs targeting inflammatory factors like TNF-α and IL-6 could slow DKD, DR, and DSPN progression. Antioxidants and anti-inflammatory drugs could reduce oxidative stress and inflammation triggered by EDCs, mitigating complications. Preventive treatments are also crucial for high-risk individuals with DM. Personalized interventions, such as early detection and regular monitoring of EDC exposure, could help identify potential risks for DmiVD. Combining targeted drugs or metabolic-regulating medications for early intervention could reduce disease incidence and slow progression. Additionally, understanding EDCs’ mechanisms in DmiVD could lead to vaccine development to target immune or inflammatory responses, enhancing immune defense and preventing disease onset. Specifically, vaccines that counteract the chronic inflammation caused by EDCs could reduce the risk of diabetes and its complications.

In summary, our research provides a significant theoretical basis for the development of treatment strategies targeting the complex relationship between EDCs and DmiVD. By employing a multi-pronged approach, including targeted therapies, early interventions, and vaccine development, we can not only effectively slow the progression of DmiVD but also offer new preventive and therapeutic options in public health, advancing global diabetes prevention and control efforts.

## Conclusion

This research integrates network toxicology, molecular docking, and bioinformatics to advance the study of EDCs. It provides new insights into the pathogenic mechanisms of EDCs in DmiVD (DKD, DR, and DSPN) development. Future strategies targeting EDCs, such as developing drugs to eliminate EDCs or inhibit their effects, may offer novel approaches for preventing and treating DmiVD (DKD, DR, and DSPN).

## Data Availability

No datasets generated during and/or analyzed during the current study are publicly available.
